# Carcass and Offal Yields of Farmed Common Eland (*Taurotragus oryx*) Males, as Affected by Age and Immunocastration

**DOI:** 10.3390/ani12212893

**Published:** 2022-10-22

**Authors:** Tersia Needham, Abubakar Sadiq Musa, Radim Kotrba, Francisco Ceacero, Louwrens Christiaan Hoffman, Nicole Lebedová, Daniel Bureš

**Affiliations:** 1 Department of Animal Science and Food Processing, Faculty of Tropical AgriSciences, Czech University of Life Sciences Prague, Kamýcká 129, 165 00 Prague, Czech Republic; 2 Department of Ethology, Institute of Animal Science, Přátelství 815, 104 00 Prague, Czech Republic; 3 Center for Nutrition and Food Sciences, Queensland Alliance for Agriculture and Food Innovation (QAAFI), The University of Queensland, Digital Agricultural Building, 8115, Gatton 4343, Australia; 4 Department of Cattle Breeding, Institute of Animal Science, Přátelství 815, 104 00 Prague, Czech Republic; 5 Department of Food Quality, Faculty of Agrobiology, Food and Natural Sciences, Czech University of Life Sciences Prague, Kamýcká 129, 165 00 Prague, Czech Republic

**Keywords:** antelope, carcass yield, game meat, GnRH, immunocastration

## Abstract

**Simple Summary:**

Common eland are currently underutilized regarding their meat production potential, despite their nutritious meat. Due to their temperament and adaptability, they are well-represented in commercial farms internationally, for both consumptive and ecotourism purposes. Immunocastration has been utilized as a management strategy in mixed-sex eland herds but its effects on the slaughter performance and cutting yields of common eland males have not yet been described. Furthermore, the ideal age of slaughter for eland males culled out of breeding herds relative to edible carcass product yields has not yet been explored, as it is almost impossible to control the confounding factors under current extensive production conditions within southern Africa. The Common Eland Research Facilities at The Czech University of Life Sciences Prague allows for studies under controlled conditions, with animals which have extensive records. The results indicated that while immunocastration had no influence, age affected the offal yields, primal cut, and dissection yields, as well as the high-value: low-value meat of the carcass, with the latter being in favor of juveniles. However, sub-adult males generated higher individual muscle yields.

**Abstract:**

This study investigated the effects of immunocastration and slaughter age on the carcass yield performance of intensively farmed common eland males. Eighteen male eland (five immunocastrated juveniles, three intact juveniles, five immunocastrated sub-adults, and five intact sub-adults) were finished for four months, after which their carcass, offal, primal cut, and individual muscle yields were determined. Juveniles were ~6 months of age at the start of the experiment, while sub-adults were ~1.5 years old. Primal cuts were dissected to determine the percentage yields of meat, bones (with tendons), separable fat, and trimmings. Immunocastration had no effect on offal or carcass yields. While sub-adults had increased individual muscle yields, juveniles had greater proportionate yields of primal cuts and a greater total high-value: low-value meat ratio. Whilst slaughtering younger male eland could provide additional economic advantages, this should be considered being against changing marketing trends.

## 1. Introduction

The private wildlife/game animal industry in southern Africa has shown immense growth over the past decades, thanks to legislation supporting the ownership of wildlife and the multipurpose use of these animals to generate revenue through ecotourism, hunting activities, and culling for meat production. As a result of these activities, the game industry employs approximately twice the amount of people per unit area than livestock farms in southern Africa and contributes towards food security by providing game meat and offal derived from trophy-hunted animals to their employees and local communities [1]. Of the various economic activities conducted on game farms, hunting for trophies and meat (so-called “biltong” hunters) provides the largest source of revenue, with up to 50% of game farms depending on trophy hunting as their primary source of income [1].

However, the hunting industry faces increasing challenges, including public resistance against the practice, particularly on social media, and the sudden decline in international hunters and tourists able to visit southern Africa game farms since 2019 due to restrictions put in place against the transmittance of SARS-CoV-2 (COVID-19). According to van der Merwe, Saayman, and Jacobs [2], the impact of COVID-19 on the private game industry was (and will be) severe, with the average game farm recording a loss of USD 122,100 in a study period of three months during 2020 (March to May) associated with tourist or hunter cancellations, totaling an estimated USD 436.4 million loss for the private South African game industry. While the culling of game species for meat and offal production remains underutilized, interest in commercial game meat production is growing, as it generates the highest net revenue per biomass weight of all activities [3] and is likely to be further supported by the rising concerns in the other revenue-generating areas of game farming. Almost 50% of South African game farms currently utilize intensive husbandry practices [1] focused on improving animal production and controlled breeding selection, particularly for high-value game species and coat color variants [4], which also produce a surplus of subpar breeding animals for culling. Under these culling practices, more male game species are selected than females, as more females are required for breeding replacements than males. Typically, there are two opportunities to cull such animals, either after a post-weaning feeding/finishing period (from ~6 months to 1 year of age) or after an extended finishing period but before attainment of sexual maturity (~4 years old). While the ideal slaughter age in terms of yield, economy, and meat quality is well-studied in farm animals, it has not been adequately described for many game species under various production conditions, further exacerbated by the lack of records regarding birthdate or age of individual game animals.

Of the more common game species, the common eland (*Taurotragus oryx*) has been identified as a favorable species for domestication and commercial game meat production as early as the 1960s [5,6,7,8,9,10]. The common eland is also well-represented in captive herds internationally, within zoological gardens and private breeding farms. In fact, eland were introduced to Eastern Europe as early as 1892 [11] and have been bred for meat and milk production [12]. Common eland are adaptable to various environments and management structures [13,14], being “water-independent” and more tolerant to tannic vegetation than cattle [13,15,16,17,18]. Such adaptability has motivated the argument that large megafauna, such as the common eland, may be a suitable alternative to traditional livestock, thereby playing a role in addressing the effects of meat production on global warming [19]. Common eland also habituate well to intensive handling [20], facilitating more intensive production and record-keeping, further supporting its commercial meat production potential.

The meat from common eland aligns well with the expectations of the health-conscious consumer internationally, being low in intramuscular fat [21] with a higher proportion of polyunsaturated fatty acids compared to beef cattle fed the same diet [22]. Recent research has shown that common eland meat flavor resembles that of beef [23], but that its tenderness should be improved through processing techniques such as pelvic-suspension and wet-ageing before commercial sale [24]. Immunocastration has also been utilized as a management strategy in mixed-sex eland herds [20], with little effect seen on the physical and sensorial meat quality [24]. However, the effect of decreased androgen production due to immunocastration on the slaughter performance and cutting yield of common eland males has not yet been described. Thus, the aim of this study was to investigate the effects of slaughter age in intact males, as well as the use of immunocastration, on the slaughter performance and carcass cutting yields of male common eland under controlled intensive farming conditions.

## 2. Materials and Methods

### 2.1. Animals, Experimental Design, and Slaughtering

All experimental procedures conducted during this study were approved by the Institutional Animal Care and Use Committee at the Czech University of Life Sciences, Prague (Permit: CZU 20/19). The eland were selected from a breeding herd of captive-bred common eland (*Taurotragus oryx*) at the Czech University of Life Sciences’ Research Facilities in the Czech Republic (Permit: 63479_2016-MZE-17214).

Eighteen intact male eland were randomly selected from the herd, eight juveniles (~6 months old) and ten sub-adults (~1.5 years old), and maintained in two separate pens on straw bedding within a barn, based on their age grouping. Age was determined using birthdate records, and the maximum number of available male animals in the herd were used to reach this sample size. Whilst the sample size is small, this unique research facility allows for the absolute control of age, nutrition, environment, etc., which has not been possible up until now within the extensive southern African game production industry. They were fed ad libitum with a corn silage and lucerne haylage mix (48.1% dry matter, 6.8% crude protein, 17.3% acid detergent fiber, 25.9% neutral detergent fiber, and 4.0% ash), as well as meadow hay, and supplemented with a high-protein commercial pelleted cattle feed (88.1% dry matter, 17.0% crude protein, 2.9% crude fat, 6.9% crude fiber, and 5.3% ash) at a supplementation rate of 1% of their live body weight, according to Needham et al. [24]. Within each age group, five eland were immunocastrated using two doses of 2 mL Improvac (Zoetis Animal Health, Parsippany-Troy Hills, Morris County, NJ, USA) given four weeks apart, and injected subcutaneously into the area above the shoulders with a Sterimatic safety needle guard system [25]. The second vaccination was given three months prior to slaughter. As only eight juvenile males were available, three remained as non-castrated (intact) controls, and five were immunocastrated. The sub-adult group consisted of five control intact males and five immunocastrated males.

After a four-month feeding period, all eland were slaughtered using a captive bolt at the research facilities, within a squeeze-restraint system, and under the supervision of a state veterinarian fulfilling all legislative and slaughter permissions within the Czech Republic, according to the Czech Veterinary Care Act No. 166/1999 with its later amendments, and within the specific permission no. SVS/2018/100483-S for the on-farm slaughter of the eland. For the 12 h prior to slaughter, animals had ad libitum access to water and hay. Prior to entering the squeeze-restraint, each animal was weighed individually to determine the live slaughter weight. Immediately after stunning, the animal was exsanguinated through thoracic sticking and suspended to facilitate bleeding. The internal organs were removed after bleeding, placed into individual labelled bags, and transported with the carcasses (skin on, in a refrigerated truck at 4 °C) within 90 min to the abattoir at the Institute of Animal Sciences Prague, where the carcasses were further dressed. All fore and hind feet were removed at the carpus and tarsus joints, respectively, and weighed together for each animal. The rest of the carcass was then skinned, including the skin on the head and tail, and weighed. The (skinned) head was then removed between the atlas and axis vertebrae, and the tongue was removed to be weighed separately. The penis and testes (the latter as a pair within the scrotum) were weighed. The internal offal was separated into the kidneys, liver, heart, spleen, lungs, trachea, kidney fat, heart fat, stomach/omentum fat (over the rumen), and weighed individually. The rumen and reticulum were emptied and weighed together. The offal items’ weights were expressed as a percentage yield of the live weight at slaughter. The carcass was weighed, and split in half, down the spinal column, before cooling at 4 °C for 24 h. The tail was removed and weighed after carcass chilling. The dressing percentage was calculated using the hot carcass weight and live weight at the point of slaughter.

### 2.2. Primal Cutting Yields

**Figure 1:**
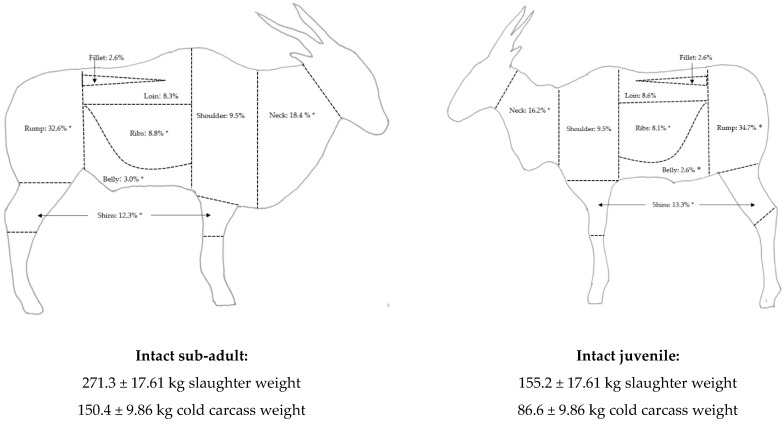
The primal cut yields (as a % of cold side weight) of sub-adult and juvenile common eland males. * indicates significant differences at *p* < 0.05.

After cooling for 24 h at 4 °C, the carcasses were weighed to determine the cold carcass weight and then randomly selected sides of each carcass were fabricated into primal cuts (Figure 1). The shanks were removed at the elbow and stifle joints, from the fore and hindquarters, respectively. The neck was removed between the last cervical vertebra and first thoracic vertebra. The *psoas major* and *minor* muscles (tenderloin) were then removed from inside the carcass. The shoulder was removed by cutting the carcass half directly behind the foreleg, in the region between the fifth and sixth thoracic vertebrae, counted from the cranial end of the carcass. The rump was removed by cutting between the lumbar vertebrae and sacral vertebrae region, in front of the pelvis. The belly was removed by cutting along the bottom edge of the ribs, and along the edge of the *longissimus lumborum* (LL) section of the *longissimus thoracis et lumborum* (LTL) muscle. The ribs were subsequently sectioned from the loin (back), cutting a straight line through them, below the vertebrae and the LTL muscle. The shanks, shoulder, rump, loin, belly, and ribs were weighed individually and expressed as a percentage yield of the cold carcass side. Each primal cut was then further dissected into muscle, bone, fat, and connective tissue, which were weighed and expressed as a percentage yield of the relevant primal cut. Subsequently, the carcass half’s meat to bone ratio were calculated, as well as the total meat yield (which included the lean meat from all joints plus the lean trimmings), connective tissue, and separable fat yields. The high-value meat yield was calculated as the total weight of the lean meat from the trimmed rump, shoulder, loin and fillet (sold fresh as whole muscles or steaks), and the low-value meat yield included the lean meat from the remaining joints plus the lean trimmings (typically processed further by grinding for products such as fresh and fermented sausages, mince/ground meat, and burgers), according to Bureš and Bartoň [26]. Thereafter, seven individual muscles were removed and weighed: LTL (including both the lumbar (LL) and thoracic (LT) sections), *biceps femoris* (BF), s*emimembranosus* (SM), s*emitendinosus* (ST), *supraspinatus* (SS), *infraspinatus* (IS) and *psoas major* (PM).

### 2.3. Statistical Analysis

Statistical analysis was performed using the SAS statistical package (SAS Institute, Cary, NC, USA). Normality of the data set was tested and confirmed with the Shapiro–Wilk’s test, and homogeneity of variance was tested and confirmed with Levene’s test. Data were analyzed with a mixed linear model, and parameters were estimated using the Restricted maximum likelihood method. The fixed effects of age and castration status, and their interactions, were included in the model analyzing the offal and carcass cutting performance (Table 1 and Table 2). As the interaction was not significant (*p* > 0.05) for the offal yields and deboning yields, they are not presented in Table 1 and Table 2. The individual muscle yields was evaluated using the fixed effects of age and muscle, and their interactions, and the random effect of animal. The interaction between muscle and yield was significant for the individual muscle yields and thus are presented in Table 3. Differences between groups were compared by Tukey’s range test. The data in the tables are presented as least squares mean and the standard error of the mean. Differences were considered significant at *p* ≤ 0.05.

## 3. Results

No interactions between the main effects of castration status and age were reported for any parameters measured. Furthermore, immunocastration had no influence on the carcass, offal, and primal cut yields. Age at slaughter influenced the slaughter weight (*p* < 0.001), and hot carcass weight (*p* < 0.001) but did not influence the dressing percentages of the carcasses (Table 1). In terms of offal yields, the feet (*p* < 0.001), kidneys (*p* = 0.031), trachea (*p* < 0.001), and empty rumen + reticulum (*p* = 0.048) contributed a larger proportionate yield of the slaughter weight in juveniles. However, the skin (*p* < 0.001), penis (*p* < 0.001), and testes (*p* < 0.001) yields were greater in sub-adults. The head, tongue, liver, heart, spleen, lungs, trachea, tail, kidney fat, heart fat, omentum fat, and empty rumen + reticulum yields were not affected by age or immunocastration.

The cold carcass side weights of sub-adults were heavier than juveniles (*p* < 0.001), but juveniles produced greater yields of high-priced meat (*p* = 0.003) and thus a better high- to low-priced meat ratio with a lower total carcass trimming yield (*p* = 0.010) (Table 2). The primary contribution to this result is the greater whole rump yield (*p* = 0.001) with a greater muscle yield (*p* = 0.006) and less trimmings (*p* = 0.009). Juveniles had a lower yield of cheaper primal cuts, such as the whole ribs cut (*p* = 0.026), which contained less separable fat (*p* = 0.030) than sub-adults, as well as a lower whole belly yield (*p* = 0.009) and whole neck yield (*p* < 0.001) (Figure 1). Juveniles had marginally greater whole shin yields compared to sub-adults (*p* = 0.018) (Figure 1), with a lower proportion of meat (*p* = 0.004) and greater proportion of bones and tendons (*p* = 0.004).

Regarding the yields of the seven individual muscles, immunocastration had no influence (*p* = 0.904) but an age-muscle interaction existed (*p* < 0.0001). Sub-adult eland had higher percentage yields for the LTL (*p* < 0.0001), BF (*p* < 0.0001), ST (*p* = 0.040), and SM (*p* = 0.0022) muscles compared to juveniles, while the IS, PM, and SS muscles did not differ (Table 3). The LTL muscle showed the highest proportional yield in the sub-adult carcasses compared to the other muscles, followed by the BF > SM > ST, IS, PM, and IS muscles. However, in the juvenile carcasses, the LTL proportional yield was similar for the LTL and BF muscles, followed by the SM > ST, IS, SS, and PM muscles (Table 3).

## 4. Discussion

The common eland males within the present study showed favorable dressing percentages, regardless of their age category, and showed superior carcass yields compared to free-ranging sub-adult common eland harvested in South Africa [27] and compared favorably to the average dressing percentages reported for sub-adult beef cattle. Currently, up to 50% of South African game ranches combine livestock and game farming [1] (often with mixed species of game). Furthermore, marginal land that was previously utilized for livestock production, especially cattle, has been transformed into game ranches [1]. Thus, the relative performance of game animals to that of livestock provides important information for producers. As shown in Table 4, European beef cattle breeds slaughtered at typical carcass weights of approximately 300 kg have dressing percentages of approximately 53% [28] depending on the breed utilized [29], as similarly reported for feedlot-fed continental breeds of cattle with dressing percentages from 54.1 to 59.1% [30]. For European-type cattle breeds under the same intensive feeding conditions and slaughtered at the same live weight (650 kg), the dressing percentages depends on their purpose (beef, dual-purpose, or dairy) and maturity type (early, medium, or late); for example: Holstein = 52.6%, Fleckvieh 55.9%; Aberdeen Angus: 56.0%; Gascon: 60.3% [29]. Free-ranging sub-adult common eland in South Africa [27] show lower dressing percentages than the farmed eland in the present study but were comparable to the dressing percentage (53.8%) of popular cattle breeds (Nguni, Bonsmara, and Angus) utilized in South Africa on natural grazing [31]. Thus, raising common eland under improved husbandry conditions can increase their dressing percentages and, thus, carcass yields, providing economic benefits depending on the carcass composition and feedlot performance (feed conversion ratio).

In comparison to other southern African antelope commonly used for meat production (Table 4), the common eland in the present study showed similar dressing percentages to sub-adult and adult gemsbok [32], whilst smaller antelope species, such as sub-adult blesbok [33], adult springbok [33], and sub-adult [33], and adult impala [34] had higher dressing percentages, despite the type of production system used. However, gut fill has a significant impact on dressing percentages, and without feed withdrawal (which has welfare implications), it is difficult to accurately compare these values, particularly so when animals are culled in the field, as done when hunting antelope extensively.

In comparison to adult fallow deer (*Dama dama*), a popular internationally farmed European game species, common eland showed similar dressing percentages, compared to fallow deer fed barley on pasture grazing in Europe (56.2%) [35], which is more favorable than fallow deer raised extensively in South Africa (47.4%; Table 4) [36]. The fallow deer carcasses within the study of Kudrnáčová et al. [35] were also dissected utilizing the same methodology as the present study to determine the carcass composition. Eland sub-adults and juveniles in the present study (farmed under controlled conditions) had greater proportionate yields of total meat and high-priced meat than fallow deer supplemented with barley [35], with less than half the proportionate yield of separable fat. Thus, eland could be considered as an alternative game meat species in this regard as well, providing leaner carcasses that could be more attractive to the health-conscious consumer.

**Table 4:** Summary of previously reported offal yields (as a % of live weight at slaughter) of different antelope species commonly used for meat production under different husbandry systems, against that of beef cattle. Data extracted from Needham et al. [27], Hoffman et al. [32], Van Zyl et al. [33], Needham et al. [34], AHDB [28], and Fitzhenry et al. [36]. Only data for male animals are summarized. Reported standard deviations are given where possible.

	Eland	Gemsbok		Blesbok	Springbok	Impala		Beef Cattle	Fallow Deer
	Free-Ranging	Free-Ranging		Free- Ranging	Free- Ranging	Free- Ranging	Semi- Extensive	Intensive	Extensive
	Sub-Adult	Sub-Adult	Adult	Sub-Adult	Adult	Sub-Adult	Adult	Sub-Adult	Adult
**Slaughter weight**	305.4 ± 23.32	101.7 ± 5.26	187.6 ± 3.86	50.4 ± 5.0	33.7 ± 4.6	26.3 ± 4.5	36.4 ± 1.30	600	47.4 ± 12.34
**Hot carcass weight**	156.9 ± 15.08	52.6 ± 3.42	96.2 ± 4.41	24.9 ± 2.2	19.4 ± 2.5	14.5 ± 2.2	21.6 ± 0.82	318	29.6 ± 7.72
**Dressing** **percentage**	50.8 ± 1.46	51.4 ± 0.76	53.3 ± 1.7	62.9 ± 1.5	64.9 ± 1.8	63.5 ± 2.9	59.1 ± 0.76	53	61.5 ± 1.47
**Head + tongue**	4.95	6.3	5.7	8.6	6.5	6.4	6.5	2.5	5.4
**Feet**	2.3	2.8	2	-	3	-	3.3	1.9	3.3
**Kidneys**	0.3	0.3	0.2	0.3	0.6	0.6	0.3	0.19	0.3
**Liver**	1.4	1.3	1	1.3	2.8	2.5	1.6	1.3	1.8
**Heart**	0.5	0.6	0.5	1.1	0.8	1	0.7	0.37	0.9
**Spleen**	0.2	0.4	0.3	0.3	0.3	0.7	0.5	0.21 *	0.3
**Lungs + trachea**	1.5	2.2	1.6	1.9	1.9	2.3	2.1	0.84	1.2 ^+^
**Skin**	6.6	6.6	7.8	5.6	5.6	5.8	5	7	6.6
**Penis**	0.08	-	-	-	-	-	0.05	-	-
**GIT (full)**	28	-	-	33.1	33.1	22.9	18.9	14.3	16.7
**Source** **reference**	**27**	**32**	**32**	**33**	**33**	**33**	**34**	**28**	**36**

* with pancreas; ^+^ without trachea.

As shown in the present study, slaughtering common eland males after a finishing period post-weaning (i.e., juveniles slaughtered at 10 months of age and 155 kg slaughter weight) produced carcass with a greater yield of high-value meat, and a more favorable high: low value meat ratio, compared to sub-adult males selected out of the breeding replacement herd (i.e., slaughtered at ~2 years old and 270 kg slaughter weight). Juvenile eland also had lower carcass trimmings compared to sub-adults in the present study, and thus lower carcass waste. Therefore, omitting the additional feeding period by slaughtering younger animals may provide more favorable economic returns when considering the optimal age of slaughter for common eland males. Primarily, the differences in the high-value meat were in the rump cut, specifically in its meat yield, which is ideal for producing high-value fresh steaks, roasts, and chops, as well as traditional South African products such as “biltong” that require large muscles from the hindquarter or loin. Biltong from various game species (various antelope and ostriches) is a common and popular product in both small and large-scale butchers and supermarkets in South Africa and Namibia. It is primarily favored for its leanness compared to beef biltong and is currently sold at a premium price of up to three times that of fresh game meat products (depending on the cuts/muscles). Bureš and Bartoň [26] compared the effect of age (14 months compared to 18 months) on the carcass traits of Charolais × Simmental cattle, utilizing a similar carcass breakdown methodology and found that younger bulls have a higher proportion of high-priced meat, primarily from the rump, yielding a higher high-value: low-value meat. They further concluded that extending the finishing period utilizing a high energy diet is not advantageous, especially considering the poorer feed conversion ratio reported during the additional four months of fattening [26].

The juvenile common eland carcasses in the present study had lower yields of low value cuts, including the ribs, belly, and neck. This is expected, as the juvenile eland males were slaughtered prior to puberty, whereafter development of the forequarter is more pronounced as a result of secondary sexual characteristics linked with increased androgen production, such as the development of a large and muscular neck, as well as increased chest girth as they approach their mature live weight [37]. These cuts (rib, belly, and neck) are either used for stewing meat (neck) or deboned and processed into traditional South African sausage products such as “boerewors” and “droëwors” for value-adding. Both boerewors and droëwors are prepared in a similar fashion, where fresh meat is trimmed, ground together with beef or lamb fat and traditional spice mixtures and stuffed into natural casings. Thereafter, boerewors is sold as a fresh sausage product (i.e., not cured, smoked, nor cooked) while droëwors is hung and dried under a temperature- and humidity-controlled environment. As the juvenile eland typically had less bones and tendons, separable fat, and trimmings in these cuts than the sub-adults, this provides further advantage when the cuts are processed for these traditional sausage products, as most connective tissue (especially tendons) are removed when preparing the meat for mincing and stuffing.

On the other hand, when considering the yields on individual muscles, the sub-adult common eland produced carcasses with higher muscle yields (in relation to their carcass weight) for all muscles analyzed in the current study. Currently, commercial marketing and sale of game meat does not always consider factors such as origin, species, sex, age, muscle type, etc. However, marked differences in meat quality, processing, and shelf-like potential have been shown in many antelope species as a result of these factors. As shown by Needham et al. [27], the meat quality of common eland meat depended on the muscle type, regardless of the sex of the animal, motivating the need to consider the muscle type when marketing fresh meat products and choosing cuts for further processing. Depending on the future development of game meat marketing strategies, producing larger individual muscles could prove to be advantageous, especially when considering that due to the low levels of intramuscular fat, seaming out individual muscles is easier in the eland than in domesticated species such as cattle. In comparison to free-ranging sub-adult eland in South Africa [27], the intensively farmed eland in the present study showed greater yields for all muscles when considering the mean yields for both age groups. In comparison to the extensively farmed South African sub-adults [27], which were slaughtered at similar live weights to those in the current study, the farmed eland show improvements in all muscle yields, particularly in high-value commercially relevant muscles such at the LTL, BF, and SM, which are often used to produce biltong [38] and may be used for production of fresh steaks (with a larger portion size) and roasts.

An often-overlooked portion of the unprocessed carcass is the edible offal yield; however, this fraction is extremely relevant to human nutrition, particularly in the diet of developing countries, such as Africa and Asia [39,40]. Edible offal includes a diverse range of organs and carcass by-products, including the heart, tongue, lungs, spleen, kidneys, feet, head, gastrointestinal tract, testes, brains, bone marrow, blood, etc. Furthermore, both the edible and non-edible portion of carcasses may be relevant for non-consumptive purposes, such as hide production, pet food, fertilizers, and traditional medicines [41,42]. Considering that offal contributes up to 40 to 50% of unprocessed game carcasses (see Table 4 for examples), the yield and value of offal should not be neglected.

Typically, game meat animals have a higher proportion of the carcass being represented by the head compared to cattle (particularly, the *Bos taurus*; Table 4), as they have large horns (frequently in both sexes, including eland). The contribution of this percentage, of course, depends on the game species, as they show diverse shapes and sizes. The proportionate yield of the heads of the sub-adults and juveniles in the present study were not significantly different; however, horn development starts early in the common eland. The sub-adult eland males in the present study had greater proportionate yields of their skins, showing benefits for the tanning industry, as well as reproductive organs (testes and penis) due to their more advanced development, while juveniles had higher proportions (albeit minor) of edible offal, such as the kidneys and empty rumen. The offal yields of the common eland in the present study were comparable to the offal yields of other game species (Table 4).

The use of immunocastration to control behavior and meat quality issues in male livestock has grown commercially over recent years, particularly within the pork industry [43], demonstrating beneficial effects on agonistic behaviors and male taints in meat. Under captive and confined conditions of intensive production systems, as for the eland in the present study, it is necessary to control indiscriminate breeding and agonistic behaviors. Thus, immunocastration could be used as a welfare-friendly tool in the husbandry of captive eland males. Due to its suppressive effect on androgen production, immunocastration may diminish the lean muscle growth potential; however, as shown in the present study, it has no effect on carcass or muscle yields. The low number of experimental units utilized, particularly for the juvenile eland, likely influenced the lack of interactions and effects of immunocastration on the parameters measured; this is the result of the total number of animals available in the experimental breeding herd. However, this study represents the first data on these factors (and under controlled conditions), which may be expanded upon in further studies.

## 5. Conclusions

The use of immunocastration did not influence the slaughter and carcass performance of young (<18 months) common eland males under intensive husbandry conditions. However, age affected the primal cut yields and individual muscle yields; slaughtering juvenile male eland after a short fattening period yielded carcasses with a greater proportion of high-value meat cuts, while slaughtering sub-adult males generated higher individual muscle yields, likely linked to their stage of body development. While omitting a longer fattening period strengthens the potential economic advantage of slaughtering younger animals, future marketing strategies regarding muscle types, game meat product developments, and game meat prices could influence this. However, the number of animals utilized in this study remain limited, and thus, future studies should consider evaluating not only a larger number of animals but also age and sex categories. Therefore, further investigation into modelling the slaughter and carcass yields of common eland relative to their sex and stage of development on the typical growth curve can allow for identification of the economically optimum age/weight of slaughter for male common eland relative to changing marketing trends.

## Figures and Tables

**Table 1:** Effect of age and castration on slaughter performance and offal yields of farmed common eland males. Significant differences are reported for the main effects, as no interactions (*p* > 0.05) between main effects were found.

	Age		Castration Status		SEM	*p*-Value	
	SA	J	NON	IC		A	C
Slaughter weight (kg)	271.3	155.2	208.6	217.9	17.61	0.0002	0.694
Hot carcass weight (kg)	153.8	89.7	120.4	123	9.66	0.0002	0.843
Dressing percentage	56.8	57.7	57.9	56.6	0.72	0.393	0.208
**Proportionate yields (% of live slaughter weight)**							
Head	3.91	3.93	4.01	3.83	0.123	0.872	0.291
Tongue	0.20	0.21	0.20	0.20	0.008	0.560	0.888
Feet	2.18	2.53	2.39	2.32	0.057	0.0004	0.361
Skin	7.68	5.88	6.84	6.71	0.295	0.0004	0.729
Kidneys	0.19	0.21	0.20	0.20	0.007	0.031	0.673
Liver	1.24	1.29	1.24	1.29	0.046	0.384	0.474
Heart	0.45	0.47	0.47	0.44	0.014	0.324	0.238
Spleen	0.15	0.15	0.15	0.14	0.008	0.816	0.394
Lungs	0.57	0.61	0.59	0.59	0.022	0.203	0.963
Trachea	0.19	0.28	0.24	0.24	0.017	0.0012	0.941
Tail	0.18	0.16	0.18	0.16	0.010	0.088	0.144
Penis	0.15	0.11	0.13	0.13	0.007	0.0006	0.763
Testes + scrotum	0.11	0.05	0.09	0.08	0.006	<0.0001	0.760
Kidney fat	0.12	0.15	0.14	0.13	0.025	0.350	0.715
Heart fat	0.36	0.30	0.34	0.32	0.050	0.397	0.831
Omentum fat	0.13	0.11	0.12	0.12	0.414	0.414	0.970
Empty rumen + reticulum	2.08	2.37	2.20	2.25	0.099	0.048	0.675

SA = sub-adult; J = juvenile; NON = intact, non-castrated male; IC = Immunocastrated; SEM = standard error of the mean; A = age; C = castration status. Bold, it improves the readability of the table and would prefer that they remain.

**Table 2:** Effect of age and castration on the carcass deboning yield of farmed common eland males. Significant differences are reported for the main effects, as no interactions between treatments (*p* > 0.05) were found.

	Age		Castration Status		SEM	*p*-Value	
	SA	J	NON	IC		A	C
Cold side weight (kg)	75.2	43.3	58.5	60	4.93	<0.001	0.832
**Proportionate yields (% of cold side weight)**							
Total meat yield	82	80.9	81.3	81.6	0.43	0.071	0.252
High- priced meat	43.8	46.5	44.8	45.5	0.59	0.003	0.415
Low-priced meat	38.3	34.7	36.5	36.2	0.37	<0.0001	0.576
Total bones	15.3	16.4	16.0	15.7	0.42	0.073	0.568
Total tendons	1.0	1.2	1.1	1.1	0.1	0.187	0.922
Total trimmings	5.0	4.2	4.7	4.4	0.2	0.010	0.334
Total separable fat	1.7	1.5	1.6	1.6	0.2	0.536	0.909
**Ratios**							
High:low-priced meat	1.146	1.355	1.237	1.264	0.023	<0.0001	0.447
Meat/bones	5.035	4.063	4.784	4.883	0.121	0.047	0.603
**Primal cut yields (% of cold side weight) and tissue dissection yield (% of primal cut weight)**							
Rump	32.6	34.7	33.9	33.4	0.39	0.001	0.378
Meat	89.6	91.0	90.0	90.6	0.34	0.006	0.220
Bones and tendons	6.8	6.2	6.7	6.3	0.27	0.165	0.318
Separable fat	0.80	0.80	0.85	0.75	0.12	0.844	0.525
Trimmings	2.90	1.90	2.43	2.33	0.24	0.009	0.761
Shoulder	9.5	9.5	9.3	9.7	0.25	0.979	0.312
Meat	84.5	87.2	84	87.8	2.49	0.422	0.266
Bones and tendons	10.4	9.7	10.9	9.3	1.05	0.645	0.272
Separable fat	2.2	1.8	2.4	1.7	0.49	0.509	0.314
Trimmings	2.8	1.2	2.8	1.3	1.05	0.264	0.290
Loin	8.3	8.6	8.5	8.4	0.30	0.550	0.828
Meat	54.9	55.6	54.1	56.4	2.03	0.812	0.400
Bones and tendons	26.8	28.3	27.5	27.5	1.47	0.465	0.987
Separable fat	2.8	0.9	2.1	1.5	0.83	0.096	0.599
Trimmings	15.4	15.3	16.3	14.5	1.10	0.923	0.230
Fillet	2.6	2.6	2.6	2.6	0.05	0.478	0.838
Meat	75.2	74.1	73.1	76.2	2.49	0.727	0.372
Separable fat	4.3	3.5	3.4	4.4	0.66	0.386	0.284
Trimmings	20.5	22.5	23.5	19.5	2.32	0.528	0.214
Ribs	8.8	8.1	8.5	8.5	0.21	0.026	0.912
Meat	63.1	63.6	64.1	62.6	1.02	0.723	0.264
Bones and tendons	24.4	26.5	25.3	25.6	0.73	0.052	0.810
Separable fat	5.30	3.60	4.43	4.46	0.55	0.030	0.961
Trimmings	7.2	6.4	6.1	7.4	0.73	0.447	0.200
Belly	3.0	2.6	2.7	2.8	0.12	0.009	0.227
Meat	59.6	61.2	62.5	58.3	2.48	0.637	0.224
Bones and tendons	19.8	20.5	20.1	20.2	1.11	0.659	0.902
Separable fat	3.2	8.5	5.2	6.5	2.77	0.171	0.723
Trimmings	17.4	9.8	12.3	14.9	2.79	0.061	0.484
Shins	12.3	13.3	12.9	12.7	0.28	0.018	0.573
Meat	70.2	66.7	68.5	68.3	0.77	0.004	0.843
Bones and tendons	29.8	33.3	31.5	31.7	0.77	0.004	0.843
Neck	18.4	16.2	17.3	17.4	0.34	<0.001	0.868

SA = sub-adult; J = juvenile; NON = intact, non-castrated male; IC = immunocastrated; SEM = standard error of the mean; A = age; C = castration status. Bold, it improves the readability of the table and would prefer that they remain.

**Table 3:** Effect of age and muscle type interaction on the individual muscle yields (as a percentage of the cold side weight) of farmed common eland males.

	Muscle Type and Animal Age															
	LTL		BF		ST		SM		IS		SS		PM		SEM	*p*-Value
	SA	J	SA	J	SA	J	SA	J	SA	J	SA	J	SA	J		
**Yield (%)**	4.58 ^a^	2.6 ^cd^	4.06 ^b^	2.36 ^d^	1.43 ^f^	0.85 ^gh^	3.08 ^c^	1.96 ^e^	1.14 ^fgh^	0.61 ^hi^	0.84 ^hi^	0.49 ^i^	1.33 ^fg^	0.67 ^hi^	0.168	<0.0001

^a–i^ Means within rows with different superscripts differ (*p* ≤ 0.05); SA = sub-adult; J = juvenile; LTL = *longissimus thoracis et lumborum*; BF = *biceps femoris;* SM = *semimembranosus*; ST = *semitendinosus*; SS = *supraspinatus*; IS = *infraspinatus*; PM = *psoas major*; SEM = standard error of the mean.

## Data Availability

The data are available from the corresponding author upon request.
